# Differentiating Keratoconus and Corneal Warpage by Analyzing Focal Change Patterns in Corneal Topography, Pachymetry, and Epithelial Thickness Maps

**DOI:** 10.1167/iovs.15-18938

**Published:** 2016-08-02

**Authors:** Maolong Tang, Yan Li, Winston Chamberlain, Derek J. Louie, Julie M. Schallhorn, David Huang

**Affiliations:** Center for Ophthalmic Optics & Lasers Casey Eye Institute, Department of Ophthalmology, Oregon Health & Science University, Portland, Oregon, United States

**Keywords:** keratoconus, optical coherence tomography, corneal topography

## Abstract

**Purpose:**

To differentiate between keratoconus and contact lens-related corneal warpage by combining focal change patterns in anterior corneal topography, pachymetry, and epithelial thickness maps.

**Methods:**

Pachymetry and epithelial thickness maps of normal, keratoconus, and warpage, and forme fruste keratoconus (FFK) eyes were obtained from a Fourier-domain optical coherence tomography (OCT). Epithelial pattern standard deviation (PSD) was calculated and combined with two novel indices, the Warpage Index and the Anterior Ectasia Index, to differentiate between normal, keratoconus, and warpage eyes. The values of the three parameters were compared between groups.

**Results:**

The study included 22 normal, 31 keratoconic, 11 warpage, and 8 FFK eyes. The epithelial PSD was normal (< 0.041) for 100% normal eyes and abnormal (> 0.041) for 100% of keratoconic eyes, 81.8% of warpage eyes, and 87.5% of FFK eyes. The Anterior Ectasia Index of normal eyes (1.66 ± 0.74) was significantly lower than that for the keratoconus eyes (17.5 ± 7.17), the warpage eyes (2.98 ± 1.69), and the FFK eyes (6.95 ± 5.86). The Warpage Index was positive in all warpage eyes and negative for all keratoconic and FFK eyes except three wearing rigid gas-permeable contact lens.

**Conclusions:**

The epithelial PSD can distinguish normal from keratoconus or warpage, but does not distinguish between these two conditions. The Anterior Ectasia Index is abnormal in keratoconus but not warpage. The Warpage Index is positive for warpage and negative for keratoconus, except in cases where keratoconus and warpage coexist. Together, the three parameters are strong tripartite discriminators of normal, keratoconus, and warpage.

Placido disc topography is an important tool in the recognition of forme fruste keratoconus (FFK),^1,2^ which is the most important risk factor for post-LASIK ectasia.^3^ However, the recognition of FFK on topographic displays, like axial power and tangential maps, is a complex exercise because FFK can manifest as many possible patterns of distortion. Several new tools have been developed to make the detection of FFK more reliable. The mean curvature (a.k.a. mean power) map has been shown to better characterize keratoconus than the conventional axial and tangential power maps.^4^ This is because the mean curvature map contains information of both the radial and azimuthal curvature changes that occur in keratoconus, but is not confounded by regular astigmatism. More recent studies have shown that corneal pachymetry^5–8^ and epithelial thickness maps^9–13^ can be more sensitive than Placido topography for keratoconus diagnosis.

On their own, these maps cannot differentiate keratoconus from other corneal pathologies with similar topographic patterns, such as contact lens-related warpage, dry eye disease, and epithelial basement membrane dystrophy. Because many LASIK candidates are contact lens wearers, the distinction between warpage and keratoconus is a common clinical challenge. The purpose of this study is to differentiate keratoconus from contact lens-related warpage by combining focal change patterns of several corneal maps: anterior topography, pachymetry, and epithelial thickness. Two novel diagnostic indices were developed to aid in the differential diagnosis of corneal conditions that confront the corneal and refractive surgeon.

## Materials and Methods

### Subjects

This prospective observational study was approved by the institutional review board of the Casey Eye Institute, Portland, Oregon, United States. This work is compliant with the Health Insurance Portability and Accountability Act of 1996 and adhered to the tenets of the Declaration of Helsinki. Normal subjects enrolled in this study were LASIK candidates who had no ocular diseases and have not been wearing contact lenses for at least 2 weeks prior to the exams. Keratoconus subjects included in this study were diagnosed clinically with the following inclusion criteria: topography characteristic of keratoconus^14^ (skewed asymmetric bow-tie, inferior steep spot, or claw patterns), KISA% index^15^ greater than 100, and best spectacle-corrected visual acuity (BSCVA) 20/25 or worse. Eyes with late keratoconic changes such as corneal scars or hydrops were excluded as they did not pose any diagnostic challenge. Keratoconus participants were subdivided into those who used rigid gas-permeable (RGP) and those who did not. There were no keratoconus participants who used soft contact lens. Contact lens warpage was defined as contact lens wearers with topographic abnormality. The topographic abnormality included inferior–superior asymmetry greater than 1.4 D or 5-mm zone irregularity index > 1.5 D on a slit-scanning topographer (Orbscan II, Bausch & Lomb, Rochester, NY, USA). The FFK cases in the study were the better eyes of asymmetric keratoconus subjects. These eyes were all KISA normal (KISA% <60) with the other eyes having keratoconus as per the prior diagnostic criteria.

### Topography and OCT

Anterior corneal topography was obtained and exported from the Orbscan II device (Bausch & Lomb, Bridgewater, NJ, USA). This system projects 40 optical slits, 20 from the right and 20 from the left, onto the cornea at a 45-degree angle. The resulting slit images were captured by a digital video camera and used to reconstruct the topography of corneal surface. The topography maps were repositioned to be centered on the pupil center. The KISA% index was calculated based on the Placido-based axial power maps from the Orbscan II. A Fourier-domain OCT system (RTVue, Optovue, Fremont, CA, USA) was used to acquire corneal pachymetry and epithelial thickness maps. The system works at an 830-nm wavelength and has a scanning speed of 26,000 axial scans per second. The depth resolution of RTVue is 5 μm (full-width-half-maximum) in tissue. The OCT scan pattern for mapping the cornea was “Pachymetry+CPwr,” which consisted of eight evenly spaced radial scans 6 mm in length. The pachymetry and epithelial thickness maps were also centered on the pupil center.

### Warpage Index and Anterior Ectasia Index

The pattern deviation (PD) map was defined as the percent deviation from the normal reference map (i.e., the average map of a healthy control group). It can be calculated from topography maps, pachymetry, or epithelial thickness maps. The detailed calculation method of the PD map have been described in a previous study.^10^ The normal reference maps were also established in that study.^10^ The epithelial pattern standard deviation (PSD) was the root-mean-square of the epithelial PD map.

**Figure 1:**
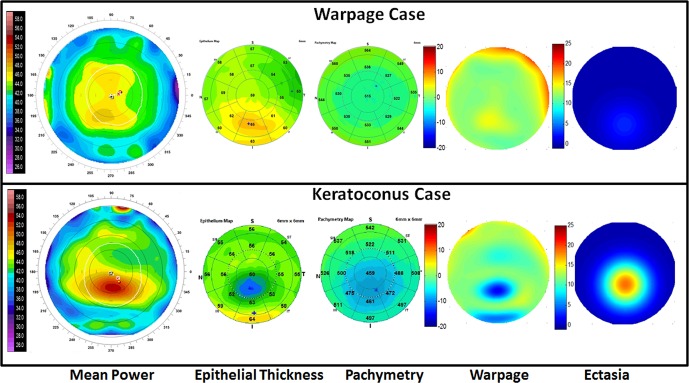
Contact lens-related warpage (*top*) and keratoconus (*bottom*) are not distinguishable by anterior topography (i.e., mean power map) when both show inferior focal steepening. They can be differentiated by the OCT epithelial map, which shows matching focal thickening in warpage and thinning in keratoconus. The pachymetry map shows focal thinning in keratoconus, but not in warpage. The warpage map is the product of the PD maps of anterior topography and epithelial thickness. The warpage map is predominantly positive (*red/yellow*) for the warpage case (*top*) and predominantly negative (*blue/green*) for the keratoconus case (*bottom*). The ectasia map is the product of fitted Gaussian waveforms for the PD maps of anterior topography and pachymetry. It shows clear cone-like pattern in keratoconus, which is absent in warpage.

The Warpage Index was designed based on the insight that anterior focal steepening is accompanied by focal epithelial thickening in contact lens-related warpage, but associated with epithelial thinning in keratoconus (Table 1). It is calculated by the dot product of the PD maps of anterior topography and epithelial thickness (Equation 1).

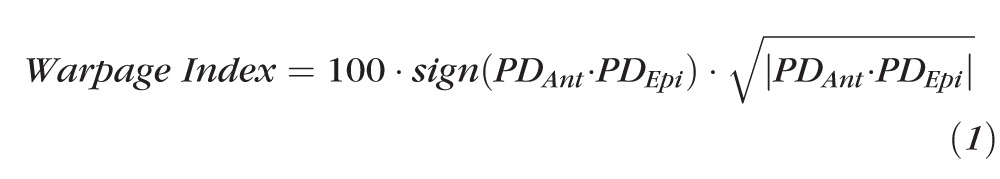
where *PD_Ant_* is the PD map of anterior mean curvature, and *PD_Epi_* is the PD map of epithelial thickness. Positive Warpage Index indicates warpage, and negative Warpage Index indicates keratoconus, as shown in Figure 1.

**Table 1:**
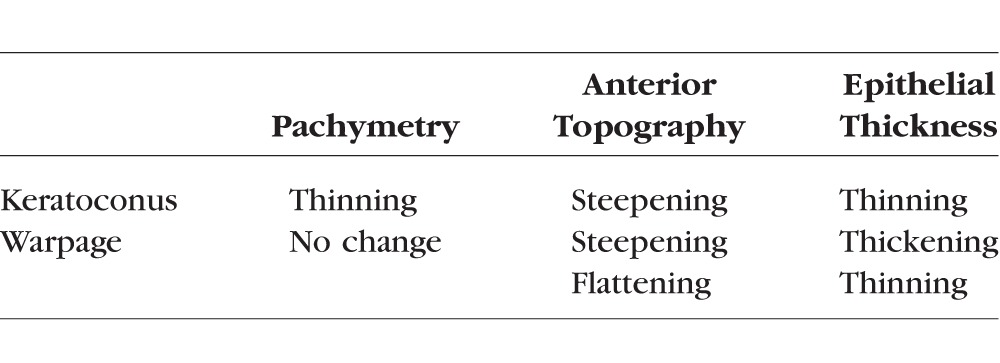
Focal Changes That Differentiate Keratoconus From Warpage on Corneal Maps

Although a negative Warpage Index was consistent with keratoconus, we wanted to incorporate the pachymetry map information to further confirm the classification. We used the Gaussian waveform, which was cone shaped, to fit the focal ectasia.^4^ The fitted Gaussian waveforms could be combined into a composite parameter using the multiplicative formula (Equation 2) to capture the coincident focal topographic steepening and pachymetric thinning (Table 1).

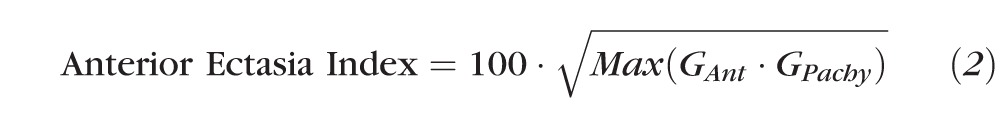
where *G_Ant_* and *G_Pachy_* are the best-fit Gaussian waveforms for the PD maps of anterior mean curvature and pachymetry. The value of the Anterior Ectasia Index is the magnitude of the combined Gaussian waveform and indicates percentage deviation from the normal reference.

### Image Processing and Statistical Analysis

Image processing was performed using MATLAB version 5.3 (Mathworks, Natick, MA, USA). Statistical analysis was performed using Excel (Microsoft Corp, Redmond, WA, USA) and SPSS 20 (IBM, Armonk, NY, USA). A generalized estimation equation model^16^ was used to account for the correlation between the eyes of the same subject. Kruskal-Wallis nonparametric tests were used to compare different groups.

## Results

The study included 31 keratoconic eyes (19 of which had recent RGP wear) of 20 subjects, 22 normal eyes of 11 subjects, 11 eyes (six eyes wearing RGP, five eyes wearing soft toric contact lenses) of eight subjects with contact lens-related corneal warpage and eight FFK eyes (four of which had recent RGP wear) of eight subjects. There was no difference in age between groups (Table 2). The keratoconus group had significantly higher steep K, topographic astigmatism, KISA%, and lower minimum pachymetry than those in normal, warpage, and FFK groups. The minimum epithelial thickness in the keratoconus group was significantly lower than that in the normal group but was not different from that in the warpage or the FFK group.

**Table 2:**
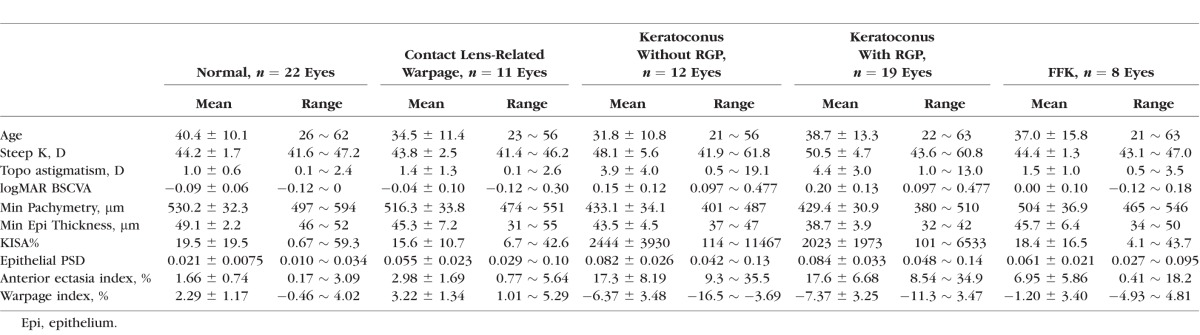
Comparison of Group Averages Among Normal, Keratoconus, Warpage, and FFK

The epithelial PSD was normal (0.021 ± 0.0075; mean ± standard deviation) for all normal eyes (100% specificity) based on a previously published diagnostic threshold of 0.041, which was 2.33 standard deviation above the mean (99 percentile of normal distribution) of 150 eyes in a normal reference group.^17^ The epithelial PSD was abnormally high for all (100% sensitivity) keratoconic (0.083 ± 0.034), 9 out of 11 (81.8% sensitivity) warpage eyes (0.055 ± 0.023), and 7 out of 8 (87.5% sensitivity) FFK eyes (0.061 ± 0.021). The epithelial PSD values for the keratoconus group, warpage group, and FFK group were all significantly (*P* < 0.001) higher than normal (Table 2). There was no difference in mean epithelial PSD values between eyes with RGP contact lens-induced warpage and eyes with soft toric contact lens-induced warpage.

**Figure 2:**
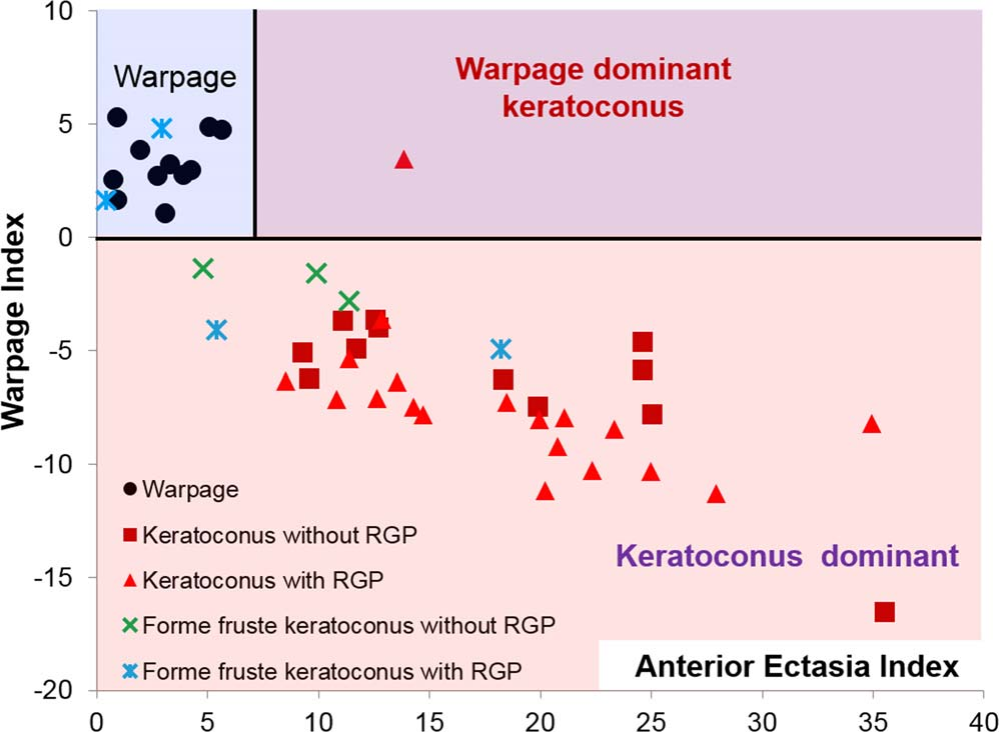
The Anterior Ectasia Index and Warpage Index can be used to differentiate warpage from keratoconus for eyes with abnormal epithelial PSD values. The *pink area* denotes keratoconus, while the *blue area* denotes warpage. The *purple area* indicates both conditions coexist.

The Anterior Ectasia Index was correlated with KISA% (Pearson's *r* = 0.60) in the keratoconus group but not in the normal (*r* = 0.14), warpage (*r* = 0.16), or the FFK group (*r* = 0.076). The Anterior Ectasia Index for the normal group (1.66 ± 0.74) was significantly lower than that for the keratoconus group (17.5 ± 7.17, *P* < 0.001), the warpage group (2.98 ± 1.69, *P* = 0.0063), and the FFK group (6.95 ± 5.86, *P* < 0.001). Using Anterior Ectasia Index of 6.92, 2.33 standard deviation above the mean (99 percentile of normal distribution) of the warpage group as the cutoff, there was 100% sensitivity and specificity in detecting keratoconus. Four of the FFK eyes had abnormally high Anterior Ectasia Index (Fig. 2).

**Figure 3:**
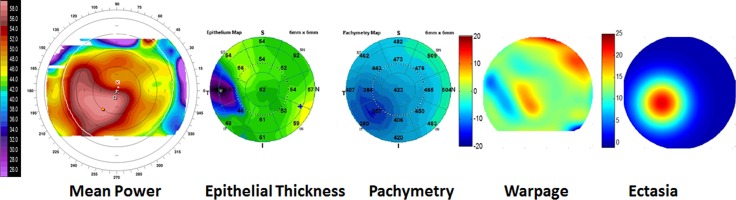
A keratoconus case that also shows signs of contact-lens related warpage.

The Warpage Index was positive in all warpage eyes (3.22 ± 1.34) and all (2.29 ± 1.17) except one normal eye. The Warpage Index was negative for all (−6.98 ± 3.32) except one keratoconus eyes (Fig. 2). The one keratoconus eye with positive Warpage Index was a RGP wearer as shown in Figure 3. The keratoconus with RGP group tended to have a slightly more negative Warpage Index (−7.37 ± 3.25) than that in keratoconus without RGP group (−6.37 ± 3.48), but the difference was not statistically significant (*P* = 0.78). Among the seven FFK eyes with abnormal epithelial PSD, five had negative Warpage Index values. The other two had positive Warpage Index and were both RGP contact lens wearers (Fig. 2).

**Figure 4:**
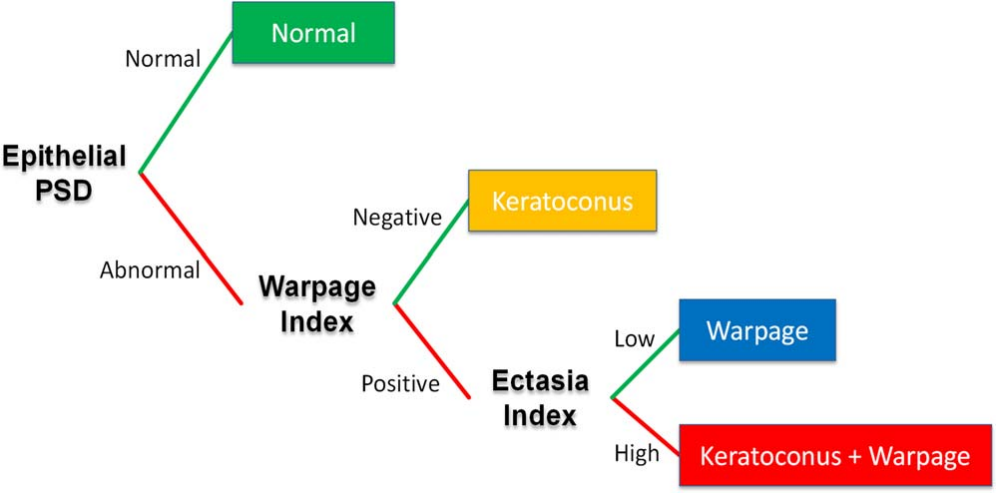
Proposed approach for comprehensive keratoconus and warpage diagnosis in a clinical setting.

Given that contact lenses are often used for vision correction in keratoconus, an overall diagnostic scheme is needed to account for this overlap. We propose the use of a decision tree (Fig. 4) that starts with the epithelial PSD, which has the highest accuracy for separating normal from abnormal corneas. The pathologic cases with abnormally high epithelial PSD are then tested with the Warpage Index. Those with negative Warpage Index values are diagnosed with keratoconus, while those with positive Warpage Index are diagnosed with warpage. The warpage cases are then tested with the Anterior Ectasia Index, with the result that the subthreshold cases has pure warpage, while the supra-threshold are diagnosed with both keratoconus and warpage. Using this scheme, all of the normal (100% specificity) and keratoconic eyes (100% sensitivity) were correctly classified. Nine of the contact lens-related warpage cases were correctly classified (81.8% sensitivity), while two were misclassified as normal. One of the 19 RGP-corrected keratoconus eyes had mixed keratoconus plus warpage pattern, while in the other 18 the keratoconus pattern predominated. Five out of 8 FFK eyes (62.5% sensitivity) were correctly classified. The three misclassified FFK eyes included one having normal epithelial PSD (misclassified as normal) and two wearing RGP contact lenses (misclassified as warpage).

## Discussion

Corneal topography is currently an essential part of the LASIK preoperative work-up to detect FFK and keratoconus. However, topography is not sensitive to very early stages of keratoconus when the topographic steepening is masked by focal epithelial thinning.^9^ Furthermore, contact lens-related warpage can sometimes manifest as inferior steepening on topography that can be indistinguishable from keratoconus or FFK.

We previously developed diagnostic parameters based on OCT corneal pachymetry and epithelial thickness maps to detect early keratoconus.^6,10^ We found that the epithelial PSD was the most accurate parameter at differentiating keratoconus from normal eyes. In 50 subclinical (corrected distance visual acuity 20/20 or better) keratoconus and 150 normal control eyes, the sensitivity was 96% at 100% specificity.^17^ Furthermore, epithelial PSD can detect abnormality in KISA-normal FFK eyes.^17^ Though the epithelial PSD is very sensitive at detecting the focal epithelial thinning that masks early ectasia on anterior topography, it is also very sensitive at detecting the uneven epithelium in contact lens-related warpage and other corneal surface distortions. To specifically diagnose keratoconus, combining pattern analysis of focal changes in different maps is needed, as has been pointed out in the global consensus definition of keratoconus and ectasia.^18^

In this study, we developed the two novel indices, Anterior Ectasia Index and Warpage Index, to differentiate keratoconus from warpage by combining the focal changes in anterior corneal topography, pachymetry, and epithelial thickness maps. To date, all keratoconus diagnostic algorithms only attempt to distinguish keratoconus from normal eyes. Our new approach is more closely tailored to the real-world application where a surgeon must distinguish between several different conditions that require different treatment decisions. An abnormally high Anterior Ectasia Index is the result of the coincident focal topographic steepening and pachymetric thinning, which is typical in keratoconus and other ectasia (i.e., pellucid marginal degeneration and post-LASIK ectasia) but not in warpage (Table 1). On the other hand, an abnormal (i.e., positive) Warpage Index is due to focal topographic steepening and flattening due to focal epithelial thickening and thinning. It is interesting to note that most normal eyes have a small positive Warpage Index. This implies that there are some naturally occurring “warpage” in normal eyes. We speculate that it might be caused by upper lid pressure molding the epithelial thickness, causing a normal pattern of slightly thinner superior epithelium and slightly flatter superior topography.^10^ Contact lens wear puts uneven pressure on the epithelium and produces more unpredictable warpage patterns. All warpage cases in our study were induced by RGP or soft toric contact lenses instead of regular soft spherical lenses, probably because RGP and soft toric contact lenses had more effect in changing the corneal epithelium.^19^ Although we did not study dry eye and epithelial basement membrane dystrophy in this paper, these conditions should also produce uneven epithelium and increase both the epithelial PSD and Warpage Index.

Using just one of the two new indices is not sufficient to distinguish between keratoconus and warpage. Though the Anterior Ectasia Index can separate the keratoconus and normal group perfectly, it does not differentiate between the warpage and normal group. Similarly, though Warpage index is positive in all warpage eyes and normal in most keratoconus eyes, it fails in cases where keratoconus and warpage coexist. Tripartite classification between normal, warpage, and ectasia requires using both new indices together with the epithelial PSD.

In most keratoconic eyes wearing RGP contact lenses, there was an abnormally high Anterior Ectasia Index and a negative Warpage Index. This is contrary to the positive Warpage Index we see in nonkeratoconic contact lens warpage. Furthermore, the keratoconus/RGP eyes tend to have more negative Warpage Index values than keratoconic eyes without contact lenses (Fig. 3). This is probably because epithelium at the cone peak comes into contact with the RGP contact lenses, resulting in epithelial thinning at a location of topography steepening—opposite of the usual warpage pattern where epithelial thinning is associated with focal topographic flattening. In the one keratoconus/RGP case where the Warpage Index was positive (Fig. 4), the cone apex was off-center inferotemporally, and the RGP-related warpage caused focal epithelium thickening that shifted the location of topographic steepening superonasally toward the central cornea. Overall, in RGP-wearing keratoconus eyes, there is a paradoxical negative shift of the Warpage Index due to cone-apex RGP touch, except in the unusual case where the RGP-corneal contact is not at the cone apex.

The main limitation of this study is that the number of cases is relatively small, which can be addressed in a future study with more cases. Another limitation is that if keratoconic distortion is extremely subtle (e.g., FFK), contact lens wear in these cases can lead to misclassification as warpage. If so, contact lens cessation will remain necessary for our classification scheme to accurately distinguish between keratoconus and warpage. Additionally, the OCT scans used in this study only covered central 6-mm diameter corneal area. This limited its ability to detect peripheral corneal abnormalities.

In summary, this study confirms that OCT-based epithelial PSD can detect corneal distortions with high sensitivity and specificity. The novel Anterior Ectasia Index and Warpage Indices can be used to distinguish between keratoconus and warpage. Combination of the three parameters provides a comprehensive diagnostic classification system to help clinicians make the appropriate diagnosis.

## References

[1] Maguire LJ, Lowry JC. Identifying progression of subclinical keratoconus by serial topography analysis. *Am J Ophthalmol*. 1991 ; 112: 41– 45. 1882920 10.1016/s0002-9394(14)76210-5

[2] Li X, Rabinowitz YS, Rasheed K, Yang H. Longitudinal study of the normal eyes in unilateral keratoconus patients. *Ophthalmology*. 2004 ; 111: 440–446. 15019316 10.1016/j.ophtha.2003.06.020

[3] Randleman JB, Russell B, Ward MA, Risk factors and prognosis for corneal ectasia after LASIK. *Ophthalmology*. 2003 ; 110: 267–275. 12578766 10.1016/S0161-6420(02)01727-X

[4] Tang M, Shekhar R, Miranda D, Huang D. Characteristics of keratoconus and pellucid marginal degeneration in mean curvature maps. *Am J Ophthalmol*. 2005 ; 140: 993–1001. 16376641 10.1016/j.ajo.2005.06.026

[5] Ambrosio R, Jr Caiado AL, Guerra FP, Novel pachymetric parameters based on corneal tomography for diagnosing keratoconus. *J Refract Surg*. 2011 ; 27: 753–758. 21800785 10.3928/1081597X-20110721-01

[6] Li Y, Meisler DM, Tang M, Keratoconus diagnosis with optical coherence tomography pachymetry mapping. *Ophthalmology*. 2008 ; 115: 2159–2166. 18977536 10.1016/j.ophtha.2008.08.004 PMC2652571

[7] Mihaltz K, Kovacs I, Takacs A, Nagy ZZ. Evaluation of keratometric, pachymetric, and elevation parameters of keratoconic corneas with pentacam. *Cornea*. 2009 ; 28: 976–980. 19724217 10.1097/ICO.0b013e31819e34de

[8] Kovacs I, Mihaltz K, Nemeth J, Nagy ZZ. Anterior chamber characteristics of keratoconus assessed by rotating Scheimpflug imaging. *J Cataract Refract Surg*. 2010 ; 36: 1101–1106. 20610086 10.1016/j.jcrs.2009.12.046

[9] Reinstein DZ, Archer TJ, Gobbe M. Corneal epithelial thickness profile in the diagnosis of keratoconus. *J Refract Surg*. 2009 ; 25: 604–610. 19662917 10.3928/1081597X-20090610-06

[10] Li Y, Tan O, Brass R, Corneal epithelial thickness mapping by Fourier-domain optical coherence tomography in normal and keratoconic eyes. *Ophthalmology*. 2012 ; 119: 2425–2433. 22917888 10.1016/j.ophtha.2012.06.023 PMC3514625

[11] Kanellopoulos AJ, Asimellis G. OCT corneal epithelial topographic asymmetry as a sensitive diagnostic tool for early and advancing keratoconus. *Clin Ophthalmol*. 2014 ; 8: 2277–2287. 25429197 10.2147/OPTH.S67902 PMC4242699

[12] Reinstein DZ, Archer TJ, Urs R, Detection of keratoconus in clinically and algorithmically topographically normal fellow eyes using epithelial thickness analysis. *J Refract Surg*. 2015 ; 31: 736–744. 26544561 10.3928/1081597X-20151021-02 PMC5357464

[13] Kanellopoulos AJ, Aslanides IM, Asimellis G. Correlation between epithelial thickness in normal corneas, untreated ectatic corneas, and ectatic corneas previously treated with CXL; is overall epithelial thickness a very early ectasia prognostic factor? *Clin Ophthalmol*. 2012 ; 6: 789–800. 22701079 10.2147/OPTH.S31524 PMC3373227

[14] Binder PS, Lindstrom RL, Stulting RD, Keratoconus and corneal ectasia after LASIK. *J Cataract Refract Surg*. 2005 ; 31: 2035–2038. 16412891 10.1016/j.jcrs.2005.12.002

[15] Rabinowitz YS, Rasheed K. KISA% index: a quantitative videokeratography algorithm embodying minimal topographic criteria for diagnosing keratoconus. *J Cataract Refract Surg*. 1999 ; 25: 1327–1335. 10511930 10.1016/s0886-3350(99)00195-9

[16] Liang KY, Zeger SL. Longitudinal data analysis using generalized linear models. *Biometrika*. 1986 ; 73: 13–22.

[17] Li Y, Chamberlain W, Tan O, Subclinical keratoconus detection by pattern analysis of corneal and epithelial thickness maps with optical coherence tomography. *J Cataract Refract Surg*. 2016 ; 42: 284–295. 27026454 10.1016/j.jcrs.2015.09.021 PMC4827714

[18] Gomes JA, Tan D, Rapuano CJ, Global consensus on keratoconus and ectatic diseases. *Cornea*. 2015 ; 34: 359–369. 25738235 10.1097/ICO.0000000000000408

[19] Wang X, McCulley JP, Bowman RW, Cavanagh HD. Time to resolution of contact lens-induced corneal warpage prior to refractive surgery. *CLAO J*. 2002 ; 28: 169–171. 12394539 10.1097/01.ICL.0000018042.02034.AB

